# Ultra high performance liquid chromatography tandem mass spectrometry for rapid analysis of trace organic contaminants in water

**DOI:** 10.1186/1752-153X-7-104

**Published:** 2013-06-18

**Authors:** Tarun Anumol, Sylvain Merel, Bradley O Clarke, Shane A Snyder

**Affiliations:** 1 Department of Chemical & Environmental Engineering, University of Arizona, 1133 E James E Rogers Way, Harshbarger 108, Tucson, AZ, 85721-0011, USA; 2 School of Applied Sciences, RMIT University, 100 LaTrobe St, Melbourne, 3000, Australia

**Keywords:** Trace organic contaminant, Pharmaceutical, Personal-care product, Glucocorticoid, PFC, Solid-phase extraction, Ultra-high performance liquid chromatography, Tandem mass spectrometry, Water quality

## Abstract

**Background:**

The widespread utilization of organic compounds in modern society and their dispersion through wastewater have resulted in extensive contamination of source and drinking waters. The vast majority of these compounds are not regulated in wastewater outfalls or in drinking water while trace amounts of certain compounds can impact aquatic wildlife. Hence it is prudent to monitor these contaminants in water sources until sufficient toxicological data relevant to humans becomes available. A method was developed for the analysis of 36 trace organic contaminants (TOrCs) including pharmaceuticals, pesticides, steroid hormones (androgens, progestins, and glucocorticoids), personal care products and polyfluorinated compounds (PFCs) using a single solid phase extraction (SPE) technique with ultra-high performance liquid chromatography coupled to tandem mass spectrometry (UHPLC-MS/MS). The method was applied to a variety of water matrices to demonstrate method performance and reliability.

**Results:**

UHPLC-MS/MS in both positive and negative electrospray ionization (ESI) modes was employed to achieve optimum sensitivity while reducing sample analysis time (<20 min) compared with previously published methods. The detection limits for most compounds was lower than 1.0 picogram on the column while reporting limits in water ranged from 0.1 to 15 ng/L based on the extraction of a 1 L sample and concentration to 1 mL. Recoveries in ultrapure water for most compounds were between 90-110%, while recoveries in surface water and wastewater were in the range of 39-121% and 38-141% respectively. The analytical method was successfully applied to analyze samples across several different water matrices including wastewater, groundwater, surface water and drinking water at different stages of the treatment. Among several compounds detected in wastewater, sucralose and TCPP showed the highest concentrations.

**Conclusion:**

The proposed method is sensitive, rapid and robust; hence it can be used to analyze a large variety of trace organic compounds in different water matrixes.

## Background

The environmental occurrence of pharmaceuticals, steroid hormones, pesticides and personal-care products, collectively termed as trace organic contaminants (TOrCs) or contaminants of emerging concern (CECs), has been consistently reported for over a decade [1-4]. The recalcitrance of certain TOrCs and their ability to pass through conventional drinking water treatment trains has necessitated frequent monitoring of these chemicals [5-7]. While the effects of many TOrCs on public health remains largely unknown, studies have shown that some of these contaminants can have drastic effects on aquatic organisms at concentrations present in wastewater [8,9]. In addition, other studies have demonstrated that a combination of TOrCs can have synergistic effects on some organisms [9,10].

Numerous studies have focused on the analysis of estrogens, both natural and synthetic [11-13], but relatively less literature is available on the occurrence and analysis of other endocrine disruptors (glucocorticoids, progestins and androgens) in aquatic environments. Glucocorticoid receptor-active compounds (GRs) are known to control inflammation and infections and hence both natural and synthetic GRs have been used to prevent swelling, asthma and other diseases in humans [14]. This increased use combined with the fact that most GRs are poorly adsorbed in the human body and quickly excreted has led to their recent detection in wastewater and surface waters worldwide [14-16]. In this work we expanded the list of steroids typically included for analysis to include an androgen (testosterone), progestins (norethisterone and norgestrel) along with several GRs.

Polyfluorinated compounds (PFCs) are a relatively new sub-class of compounds within the TOrC classification. These compounds are synthetically produced and have a wide-range of applications, including in non-stick cookware, stain-resistant carpets, and surfactants among other things [17]. This frequent usage along with their inherent biological and chemical stability make PFCs persistent in the environment and frequently detected in water [17,18], biosolids [19] and biological matrices [20]. Consequently, the two most commonly used PFCs (PFOA and PFOS) are on the USEPA’s Contaminant Candidate List 3 [21]. In addition, bioaccumulation properties, potential carcinogenicity and recent reports on toxic effects to animals [22,23] have led to the voluntary reduction in usage of PFOA and the banning of PFOS in Europe [24]. However, these two compounds are progressively being replaced by shorter chain (C < 7) PFC’s [25], of which far less is known with regards to toxicity and occurrence data. Accordingly, this study set out to include six PFCs with C4-C16 carbon chain length.

Over 82,000 chemicals are registered for industrial use in the US and the number is rapidly increasing [26]. Monitoring each chemical is not feasible; hence the significance of selecting ‘indicator’ compounds that encompass the various classes of TOrCs is critical. Recent studies have sought to identify indicator TOrCs based on their occurrence and attenuation in the environment [27]. This study selected 36 disparate compounds across seven classes of TOrCs for analysis using a single extraction method and short analysis time.

As the number of environmental contaminants monitored continues to increase rapidly, the need for reliable analytical methods offering selectivity, sensitivity and reproducibility also has increased. Over the years, numerous methods relying on a variety of instruments were developed to measure TOrCs. For instance, gas chromatography has been used to analyze volatile compounds and pesticides as well as some polar compounds and steroids using derivatization agents [28,29]. However, these techniques are time-consuming, labor intensive and limited to the analysis of compounds that are volatile and not thermally-labile.

Liquid chromatography methods have proved more effective in analyzing TOrCs. While methods using UV [30,31] and fluorescence [32,33] detectors have been proposed, methods using both single quadrupole [34,35] and triple quadrupole [36,37] mass spectrometers have been most common. However, the vast majority of these methods consider only specific classes of pharmaceuticals [38,39] or compounds with similar polarities and/or use numerous extraction methods that are time-consuming and labor-intensive. Only few methods use a single extraction procedure while still analyzing a wide variety of these compounds [29,40,41]. With the introduction of ultra-high performance liquid chromatography (UHPLC), it is now possible to operate at extremely high pressures with much smaller particle sizes which allows for rapid separation of analytes while also improving resolution and sensitivity.

This study aims to provide a simple, rapid, sensitive and robust method for the targeted analysis of 36 compounds (Table 1) representative of several TOrC classes usually considered by water utilities and regulatory agencies. The method includes several different classes of TOrCs including less studied substances like GRs and PFCs. The application of UHPLC allows for a significant reduction in sample runtime while providing good analytical separation compared to previously published methods and also providing very low ng/L detection limits in water. The proposed method includes the addition of 19 stable isotopically labeled compounds to increase accuracy and precision. This method was successfully applied to groundwater, surface water and wastewater matrices.

**Table 1:** Target compounds with use and class

**Compound**	**Use**	**Class**
Atrazine	Pesticide	Pesticide
Benzophenone	UV Blocker	Personal Care Product
Bisphenol A	Plasticizer	Industrial compound
Caffeine	Stimulant	Personal Care Product
Carbamazepine	Anticonvulsant	Pharmaceutical
N,N-Diethyl-meta-toluamide (DEET)	Insect Repellant	Personal Care Product
Dexamethasone	Anti-Inflammatory	Glucocorticoid
Diclofenac	Anti-arthritic	Pharmaceutical
Diltiazem	Antiarrhythmic	Pharmaceutical
Diphenhydramine	Anti-histamine	Pharmaceutical
Fluoxetine	Anti-Depressant	Pharmaceutical
Gemfibrozil	Anti-Cholesterol	Pharmaceutical
Hydrocortisone (Cortisol)	Anti-inflammatory	Glucocorticoid
Ibuprofen	Analgesic	Pharmaceutical
Meprobamate	Anti-anxiety	Pharmaceutical
Naproxen	Analgesic	Pharmaceutical
Norethisterone	Contraceptive	Progestin
Norgestrel	Contraceptive	Progestin
Perfluoro butanoic acid (PFBA)	Fluorosurfactant	Polyfluorinated Compound
Perfluoro butane sulfonate (PFBS)	Fluorosurfactant	Polyfluorinated Compound
Perfluoro decanoic acid (PFDA)	Fluorosurfactant	Polyfluorinated Compound
Perfluoro hexadecanoic acid (PFHxDA)	Fluorosurfactant	Polyfluorinated Compound
Perfluoro octanoic acid (PFOA)	Fluorosurfactant	Polyfluorinated Compound
Perfluoro octane sulfonate (PFOS)	Fluorosurfactant	Polyfluorinated Compound
Prednisone	Anti-inflammatory	Glucocorticoid
Primidone	Anticonvulsant	Pharmaceutical
Simazine	Herbicide	Pesticide
Sucralose	Artificial Sweetener	Personal Care Product
Sulfamethoxazole	Antibiotic	Pharmaceutical
Tris (2-chloroethyl) phosphate (TCEP)	Flame retardant	Industrial compound
Tris (2-chloropropyl) phosphate (TCPP)	Flame retardant	Industrial compound
Testosterone	Androgen	Androgen
Triamcinolone	Synthetic corticosteroid	Glucocorticoid
Triclocarban	Antibiotic	Personal Care Product
Triclosan	Anti-microbial	Personal Care Product
Trimethoprim	Antibiotic	Pharmaceutical

### Experimental

#### Chemicals and reagents

All standards and reagents used during the study were of the highest purity commercially available (≥97% for all compounds). All native standards were procured from Sigma-Aldrich (St. Louis, MO) except perfluorohexadecanoic acid (PFHxDA) from Matrix Scientific (Columbia, SC); meprobamate from Cerilliant (Round Rock, TX); and triclosan from Alfa Aesar (Ward Hill, MA). Labeled standards were purchased from Cambridge Isotope Laboratories (Andover, MA) except ^13^C_4_-PFOA, ^13^C_4_-PFOS, ^13^C_2_-PFHxA, ^13^C_4_-PFBA from Wellington Laboratories (Ontario, Canada); primidone-d_5_ and ^13^C_6_-diclofenac from Toronto Research Chemicals (Ontario, Canada); and gemfibrozil-d_6_ from C/D/N Isotopes (Quebec, Canada). A working stock of all native standards was prepared at 5 mg/L in pure methanol and diluted as required to obtain the desired concentration of calibration standards. A mix of all isotopically labeled surrogates at 1 mg/L in pure methanol was also prepared and used to spike all samples before extraction. These two solutions were stored in the dark at −20°C and new working stocks were prepared every two months. Both stocks were injected routinely on the mass spectrometer and signal response was monitored for each compound to determine if there was any degradation with time.

All solvents were of the highest purity available and suitable for LC-MS analysis. Methanol (HPLC grade), MTBE (HPLC grade), formic acid (LC/MS grade) and ammonium hydroxide (ACS grade) were obtained from Fisher Scientific (Pittsburgh, PA), while acetonitrile and ultrapure water (both HPLC grade) were obtained from Burdick and Jackson (Muskegon, MI).

#### Sample collection and preservation

Grab samples were collected from four full-scale water treatment plants across the United States. In addition, multiple samples from two surface waters and a groundwater from Tucson, Arizona were analyzed. Samples (1 L each) were collected in silanized amber glass bottles containing 50 mg of ascorbic acid to quench residual chlorine and 1 g of sodium azide to prevent microbial activity. Samples were sent to the laboratory in coolers containing icepacks and filtered through a 0.7 μm glass filter (Whatman, England) immediately upon arrival. Then, samples were stored in darkness at 4°C and extracted within 14 days. Sample preservation techniques were comparable to those previously published by Vanderford *et al.*[42].

#### Solid-phase extraction

All samples were spiked with 19 isotopically labeled surrogate standards at concentrations varying from 50 to 200 ng/L depending on analytical sensitivity and matrix type. Samples were then extracted using an AutoTrace 280 automated SPE system from Dionex (Sunnyvale, CA) using 200 mg hydrophilic-lipophilic balance (HLB) cartridges (Waters Corporation; Millford, MA). Cartridges were first preconditioned with 5 ml of MTBE, followed by 5 ml of methanol and 5 ml of ultrapure water. Samples were then loaded at 15 ml/min onto the cartridges which were subsequently rinsed with ultrapure water and dried under nitrogen flow for 30 min. While 1 L samples were collected, different volumes of sample were extracted based on the matrix. The analytes were then eluted with 5 ml of methanol followed by 5 ml of 10/90 (v/v) methanol/MTBE solution. The eluent was evaporated to less than 500 μl using gentle nitrogen flow and the volume was adjusted to 1 ml by addition of methanol. Final extracts were transferred into 2-mL vials and stored in darkness at 4°C until UHPLC-MS/MS analysis.

#### Liquid chromatography

Liquid chromatography was performed on 3 μL of sample extract using an Agilent 1290 binary pump (Palo Alto, CA) with metal solvent fittings for all analyses. The Agilent RRHD ZORBAX Eclipse Plus reverse phase C-18 column (2.1×50 mm) with a packing size of 1.8 μm was used to separate analytes in both the negative and positive electrospray ionization (ESI) modes. The column was maintained at a temperature of 30°C for the entire run in both modes.

**Figure 1:**
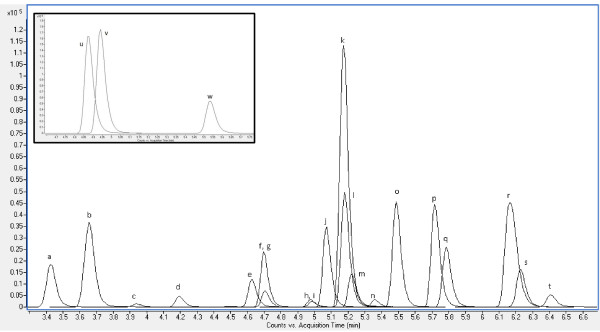
**Extracted ion chromatogram (quantifiers only) of 100 μg/L standard mixture in ESI positive. a)** caffeine, **b)** trimethoprim, **c)** sucralose, **d)** primidone **e)** sulfamethoxazole, **f)** meprobamate, **g)** triamcinolone, **h)** hydrocortisone, **i)** prednisone, **j)** simazine, **k)** carbamazepine, **l)** fluoxetine **m)** dexamethasone **n)** TCEP, **o)** atrazine, **p)** testosterone, **q)** norethisterone, **r)** TCPP, **s)** norgestrel, **t)** benzophenone, **u)** diphenhydramine, **v)** diltiazem **w)** DEET. Qualifier ion and surrogate standard chromatograms have been removed for clarity.

The mobile phase for ESI positive used two solvents comprising (A) ultrapure water with 0.1% formic acid and (B) acetonitrile with 0.1% formic acid. With a constant flowrate of 400 μl/min, solvent B was held at 5% for 1.5 min. Solvent B then linearly increased to 20% at 3 min, 45% at 4 min, 65% at 6.1, 100% at 7 min and held till 7.45 min. A post-run of 1.45 min was added to allow the column to re-equilibrate before the next analysis. This resulted in a total run-time of 9.90 min for analysis of 23 analytes (Additional file 1: Table S1 and Figure 1).

**Figure 2:**
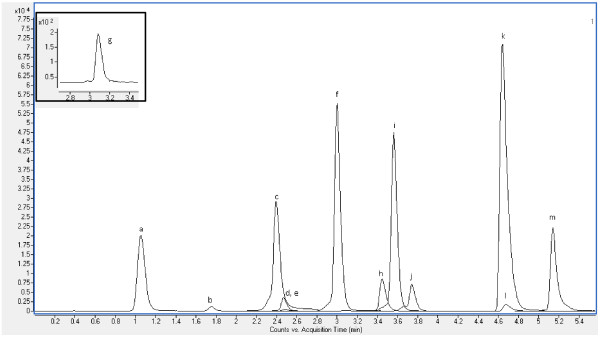
**Extracted ion chromatogram (quantifier only) of 100 μg/L standard mixture in ESI negative. a)** PFBA, **b)**naproxen, **c)** PFBS, **d)** diclofenac, **e)** Ibuprofen, **f)** PFOA, **g)** bisphenolA, **h)** gemfibrozil, **i)** PFDA, **j)** PFOS, **k)** triclocarban, **l)** triclosan, **m)** PFHxDA. Qualifier ion and surrogate standard chromatograms have been removed for clarity.

The mobile phase for ESI negative used a dual eluent system comprising (A) 5 mM ammonium acetate in ultrapure water and (B) 10/90 (v/v) water/acetonitrile with 5 mM ammonium acetate. With a constant flowrate of 400 μl/min, solvent B was linearly increased from 20% to 96% at 4.5 min and 100% at 5 min. Solvent B was held at 100% for a further 1.3 min then a post-run of 1.5 min at 20% B was added to allow the column to re-equilibrate before the next analysis. This resulted in a total run-time of 7.8 min for the analysis of 13 analytes (Additional file 1: Table S1 and Figure 2). Sample chromatograms for positive and negative ionization modes at 100 ng/mL are shown in Figures 1 and 2.

#### Mass spectrometry

Mass spectrometry was performed using an Agilent 6460 triple quadrupole mass spectrometer. Optimization was done in two steps: compound-specific and source-dependent. Initially, each compound was prepared from a neat standard at a concentration of 1 μg/ml in pure methanol and injected into the mass spectrometer at a flowrate of 500 μl/min. The first quadrupole was set to scan mode and the most intense precursor ion was selected. This was done both in positive and negative electrospray modes to select the most appropriate ion source for each compound. After the best ion source was chosen, the fragmentor voltage was optimized for each compound in scan mode. After this, the mass spectrometer was run in product ion scan (PI Scan) mode to determine the most abundant product. For this, collision energy (CE) of 20 volts was selected and then adjusted in steps of 10 to find the most abundant products. For most compounds, two transitions; a quantifier (the most abundant product) and a qualifier (the second most abundant product) were selected. Then, the mass spectrometer was set to multiple reaction monitoring (MRM) mode and the CE for each product ion was optimized. This was followed by optimization of the cell accelerator voltage (CAV); however, it was noticed that there was a possibility of cross talk between some compounds especially at low CAVs so this value was only optimized between two and seven. The analyte transitions, optimized parameters and retention times are given in Additional file 1: Table S1.

Once all the compound-specific parameters were optimized, source parameters like gas temperature, flow rate, nozzle voltage, nebulizer and capillary voltage were tuned. While, it was not possible to have optimum source parameters to suit all the compounds, best fit values were used in choosing these parameters. The source-dependent parameters for both positive and negative electrospray ionization modes are detailed in Table 2. Analysis in both ESI modes was performed using a dynamic MRM method with a delta retention time of 0.6 min for ESI positive mode and 0.8 min for ESI negative mode.

**Table 2:** Mass spectrometer source-dependent parameters

**Parameter**	**ESI Positive**	**ESI Negative**
Gas Temperature (°C)	275	225
Gas Flowrate (L/min)	11	10
Nebulizer (psi)	45	45
Sheath Gas Temperature (°C)	375	350
Sheath Gas Flowrate (L/min)	11	11
Capillary (V)	4000	3600
Nozzle Voltage (V)	0	1500
Delta EMV (V)	400	400

Data analysis and interpretation was carried out with the Agilent MassHunter software (version Rev. B.05.00). Along with monitoring the labeled isotope recoveries and the retention time, the ratio of the two transitions was also noted, which increased the accuracy of detection and reduced the possibility of false positives of the method.

#### Determination of LOD, LOQ and MRL

The instrumental limit of detection (LOD) and limit of quantification (LOQ) were determined for each compound by injecting standards at 0.02, 0.05, 0.1, 0.5, 1, 2.5, 5, 10 and 25 μg/L on the UHPLC-MS/MS system. The LOD and LOQ were defined as the concentration for which the signal to noise ratio (SNR) was greater than 3 and 10 respectively. The LOD and LOQ of all target analytes are shown in Table 3.

**Table 3:** LOD, LOQ and MRL of target analytes

**Compound**	**LOD (μg/L)**	**LOQ (μg/L)**	**Practical MRL (ng/L)**
**ESI positive**			
Caffeine	0.5	1	2.5
Trimethoprim	0.05	0.1	0.1
Sucralose	1	5	10
Triamcinolone	1	2.5	5
Primidone	1	2.5	2.5
Sulfamethoxazole	0.02	.1	.5
Meprobamate	0.1	1	2.5
Diphenylhydramine	0.02	0.1	1
Diltiazem	0.02	0.1	0.5
Hydracortisone	0.5	1	2.5
Prednisone	10	15	20
Simazine	0.1	0.5	1
Fluoxetine	0.02	0.05	0.5
Carbamazepine	0.05	0.1	0.25
Dexamethasone	0.05	0.5	1
TCEP	0.5	1	2.5
Atrazine	0.1	0.5	0.5
DEET	0.05	0.1	2.5*
Testosterone	0.5	1	1
Norethistrone	0.1	0.5	1
TCPP	0.05	1	2.5
Norgestrel	0.5	1	2.5
Benzophenone	0.02	0.5	1
**ESI negative**			
PFOA	0.02	0.5	1
PFDA	0.02	0.5	2.5
Gemfibrozil	0.05	0.5	1
PFOS	0.02	0.05	0.1
Triclocarban	0.1	0.5	1
Triclosan	0.5	2.5	5
PFHxDA	0.02	0.1	0.5
PFBS	0.02	0.05	0.5
PFBA	0.02	0.02	NA
Ibuprofen	5	10	15
Bisphenol A	1	5	15
Naproxen	0.1	1	2.5
Diclofenac	0.1	0.5	2.5

*NA* Not analyzed; * Adjusted for blank.

The method reporting limit (MRL) was determined by extracting nine samples (1 L each) of ultrapure water fortified with the target analytes at 2–3 times the LOQ (calculated from above) and spiked with isotopically-labeled surrogates. After extraction and analysis, the MRL was calculated by multiplying the standard deviation with the student’s t-test value for n-1 degrees of freedom at 99% confidence levels. The results are shown in Table 3. The method reporting limits determined were similar and in many cases lower than previously published literature [29,43].

## Results and discussion

### Chromatography

Optimization of chromatographic conditions was achieved by performing experiments with various mixtures of organic solvents and pH modifying buffers. The best mobile phase was chosen based on peak shape, peak resolution and sensitivity achieved for all compounds. Three different UHPLC reverse phase columns were also tested and the column providing the highest sensitivity for most target analytes was chosen. Details of the three columns tested are provided in Additional file 2: Table S2. Once the column and the mobile phase were selected, the gradients in both modes were optimized to achieve best separation of all target analytes while maintaining a sufficient scan speed and peak width to preserve peak shape allowing accurate integration. In addition, different injection volumes (1, 3, 5 and 10 μl) were also tested and 3 μl was used for all analysis as this gave the highest sensitivity without alteration of peak shape.

### Analyte ionization and data analysis

All but one compound were ionized by protonation[(M + H)^+^] of the uncharged molecule in the ESI positive mode. Sucralose was analyzed with the addition of a sodium adduct [(M + Na)^+^] as the [(M + H)^+^] ion was essentially absent during optimization of the compound. In the ESI negative mode, all the compounds analyzed were a result of deprotonation [(M-H)^-^] of the original neutral molecule.

The quantification of TOrCs in all samples was achieved using a calibration curve with at least nine points and an R^2^ no lower than 0.990 and typically above 0.995. All concentrations above the calibration range were diluted and re-analyzed. In a few instances, it was not possible to determine the exact concentration of an analyte due to loss of isotope signal because of dilution. In this case, concentration were reported as ‘>’ the highest calibration point. While the MRL for all TOrCs was reported in ultrapure water, this value could be impaired in other water matrices containing natural organic matter that interfere with the ionization of the analytes. To account for this, a separate MRL was determined for each sample. Initially, the lowest calibration point was chosen at or slightly above the MRL determined in ultrapure water. Using the Mass Hunter software, the expected concentrations of the calibration curve were recalculated based on the calibration equation and R^2^ using a linear regression with 1/X weighting. After comparing the calculated concentrations of all the calibration points with expected concentrations, the lowest calibration point with accuracy between 70-130% was chosen for each analyte. This value was then divided by the isotope recovery obtained for all analytes in each sample to obtain the “true” MRL in that particular sample matrix.

### Matrix spike and recoveries

Recoveries for the target analytes after extraction were determined using six replicates in three different water matrices shown in Table 4. Matrix spike levels were chosen as 100 ng/L in ultrapure water and 200 ng/L in surface water and wastewater samples. The spike recoveries were calculated by comparing this known spiking concentration with the concentration determined in unspiked samples by internal standard calibration. For ultrapure water, more than 70% of the compounds had a recovery between 90–110%. Only two compounds (diltiazem and PFHxDA) had a recovery of <70%. The recoveries in the surface water varied from 39–121% while wastewater recoveries ranged from 38–141%. While these ranges seem large, it is important to note that isotopically-labeled surrogate standards were not available for every compound. All compounds with a surrogate standard had corrected recoveries between 73–121% with the exception of diclofenac (64%) in the wastewater spike. In fact, almost all these compounds had recoveries of 85 – 115% further validating the use of isotope dilution to correct for matrix suppression and losses during SPE. The recovery of norgestrel and norethisterone were below 60% in the surface water and wastewater spike samples. Previous studies have shown that these two compounds have poor stability on storage greater than three days and this may have led to loss of analyte in the sample [44]. While every effort was made to extract the samples as soon as possible, extraction times varied between 3–14 days during this study. Spike recoveries for hydrocortisone were found to be 50% and 38% in surface water and wastewater respectively. Similar recoveries (~60%) have been seen in a previous study in wastewater [16]. To obtain better recoveries for all compounds, the use of multiple extraction procedures, and considering compound specific properties would be necessary. It was decided to proceed with this single extraction method that provided good recoveries for the majority of the compounds while allowing for significant savings in time and labor. The precision of the entire method was good as the relative standard deviation (RSD) of the replicates for almost all compounds was less than 10% in both ultrapure and surface water. While larger RSDs were observed for wastewater samples, compounds with surrogate standards were still extremely reproducible. Overall, the use of surrogate standards to correct for loss of target analytes during the extraction and analysis stages proved reliable.

**Table 4:** Matrix spike recoveries for all target analytes in three different waters

**Compounds**	**Ultrapure water**		**Surface water**		**WW effluent**	
	**Recovery (%)**	**RSD (%)**	**Recovery (%)**	**RSD (%)**	**Recovery (%)**	**RSD (%)**
**ESI positive**						
Caffeine	106	2.5	95	4.3	100	3.9
Trimethoprim	98	2.1	102	0.9	114	3.2
Sucralose	95	5.4	73	34.1	NA	NA
Primidone	97	2.9	96	1.5	113	10.0
Triamcinolone	101	4.7	48	2.3	106	4.3
Sulfamethoxazole	105	3.3	98	1.7	99	2.1
Meprobamate	97	5	74	1.5	99	8.4
Diphenylhydramine	74	5.4	94	5.4	196	3.8
Diltiazem	67	11.7	NA	NA	NA	NA
Hydracortisone	84	3.7	50	7.3	38	11.3
Prednisone	94	4.1	75	5.4	79	10.0
Simazine	99	3	73	2.0	66	2.6
Carbamezapine	101	2.1	117	1.3	98	25.5
Fluoxetine	89	5.5	97	2.3	99	5.5
Dexamethasone	91	2.7	86	2.2	88	3.4
TCEP	108	3.1	71	4.3	119	8.1
Atrazine	100	2.7	94	1.9	99	2.5
DEET	101	2.7	96	1.6	98	5.5
Testosterone	82	3.3	42	2.4	42	21.6
Norethistrone	79	2.4	39	1.9	54	2.1
TCPP	97	2.4	119	2.9	74	7.1
Norgestrel	82	3.2	57	1.3	55	6.7
Benzophenone	71	15.8	95	6.4	93	26.4
**ESI negative**						
PFBA	95	4.6	NA	NA	NA	NA
Naproxen	95	3.5	89	1.4	80	6.0
PFBS	78	7.1	111	8.0	87	3.6
Diclofenac	103	5.4	96	6.0	64	22.0
Ibuprofen	96	9.2	92	5.7	96	10.8
PFOA	101	2.3	121	6.4	115	7.4
Bisphenol A	91	15.5	97	11.6	87	10.8
Gemfibrozil	104	3.7	93	2.7	111	10.0
PFDA	97	3.6	73	15.3	65	13.4
PFOS	107	2.9	94	9.0	89	9.8
Triclocarban	105	2.8	97	1.5	107	5.0
Triclosan	74	1.1	112	2.8	141	6.7
PFHxDA	46	72.4	56	10.4	66	18.4

*NA* Not Analyzed.

### Matrix suppression

The degree of matrix suppression encountered was analyzed by comparing the instrument response (area count) of the 19 isotopically-labeled standards in the matrix spikes and samples with six instrument blanks spiked at the same concentration. The isotope recovery data in each matrix is presented in Table 5. Fluoxetine d_5_, PFBA ^13^C_4_ and diclofenac ^13^C_6_ were the only isotopically-labeled compounds to have <60% recovery in ultrapure water. The degree of suppression for most compounds increased in the wastewater matrix (250 mL) compared to the surface water (1000 mL) and ultrapure water (1000 mL) spikes even though less volume of the sample was extracted. The RSD for all analytes was below 15% and in most cases below 5%.

**Table 5:** Percent recovery of isotopically labeled standards in different water matrixes (n = 6)

**Compound**	**Ultrapure water (1000 ml)**		**Surface water (1000 ml)**		**WWTP effluent (250 ml)**	
	**Recovery (%)**	**RSD (%)**	**Recovery (%)**	**RSD (%)**	**Recovery (%)**	**RSD (%)**
Carbamezapine d_10_	77	4.4	79	2.6	70	4.9
Caffeine ^13^C_3_	79	4.7	76	3.2	56	4.5
Trimethoprim d_3_	67	4.2	66	3.1	41	6.4
Sucralose d_6_	65	5.9	31	3.3	16	4.0
Primidone d_5_	81	3.1	71	4.0	74	4.3
Sulfamethoxazole ^13^C_6_	80	3.6	30	4.6	28	6.6
Atrazine d_5_	70	3.1	59	4.1	64	3.9
Fluoxetine d_5_	40	8.3	35	6.8	40	10.7
DEET d_6_	60	9.2	70	6.8	75	10.8
PFBA ^13^C_4_	15	2.1	13	3.4	10	5.2
Naproxen ^13^C_1_d_3_	87	4.5	94	3.6	75	6.2
Diclofenac ^13^C_6_	48	1.5	30	7.5	31	14.5
Ibuprofen d_3_	86	6.5	103	4.7	90	6.5
PFOA ^13^C_4_	90	3.1	115	14.3	104	0.3
Bisphenol A ^13^C_12_	92	6.9	70	7.8	83	11.0
Gemfibrozil d_6_	86	3.1	94	4.6	117	4.9
PFOS ^13^C_4_	83	4.2	78	7.7	81	5.7
Triclocarban ^13^C_6_	61	7.5	63	3.3	54	5.7
Triclosan d_3_	122	4.5	81	3.9	68	5.1

### Blank analysis

As extremely low levels of analytes are quantified in this method, there was a possibility of contamination through various sources. Potential contamination may arise from presence of trace levels of native compound in the isotopically-labeled standards, presence of contamination in the instrument, and low-level contamination from various external sources. Initially pure methanol was injected in both ESI modes to detect the presence of any background contamination due to the solvent or instrument (Additional file 3: Figure S1 and Additional file 4: Figure S2). The target analytes were not found to be present with the exception of DEET. Next methanol blanks were fortified with the isotopically-labeled standards to determine if native compounds were introduced by the isotopes. No indication of target analyes was found in these blanks with the exception of DEET. The area counts of the DEET chromatograms present in the first two types of blanks was very similar indicating that the DEET detected was in the background and not introduced by the isotopically-labeled standard (Additional file 5: Figure S3). The concentration of DEET in the blanks was estimated using the MRL study calibration curve and subsequently the MRL for DEET was increased five times to prevent reporting of false positives. Finally, a number (n = 6) of ultrapure water samples fortified with labeled isotopes were extracted by SPE and analyzed to ensure the absence of unlabeled compounds through the extraction procedure. Further, routine fortified ultrapure water blanks were analyzed along with the samples to check for any contamination. All blanks tested during the course of the study were below MRLs.

### Occurrence in water

To demonstrate the applicability of this method, samples from three WWTPs, a drinking water treatment plant (DWTP), one ground water and two surface waters (Colorado River and Sacramento River) from around the United States were analyzed. Samples from the three wastewater treatment plants were also analyzed at different treatment points to study treatment efficacy. A summary of the treatment trains for each plant is shown in Additional file 6: Table S3. WWTP 1 served a largely urban population (approximately 500,000 people) with both domestic and industrial contribution. WWTP 2 served a considerably smaller population (approximately 17,000) with 73% of the population aged 65 or older (median age of 72 years). WWTP 3 has a capacity of approximately 70 million gallons per day (MGD) and has a predominantly domestic source of wastewater contribution. Thus, the three plants offered significantly different qualities of wastewater to be tested. DWTP 4 is an indirect potable reuse plant that receives treated wastewater effluent as its source water. The occurrence data for all 36 TOrCs at different treatment points in the four plants is shown in Table 6 along with the sample volume extracted.

**Table 6:** Occurrence of TOrCs in different water matrices

**Compound**	**WWTP 1a**			**WWTP 1b**			**WWTP 2**				**WWTP 3**				**DWTP 4**						
	**After GF**	**After AS**	**Dechlorinated final effluent**	**After AAS**	**GMF effluent**	**After UV**	**After bar screens**	**After BNROD**	**After sand filter**	**After chlorination**	**Influent**	**PostF/S**	**Post AS**	**Dechlorinated final effluent**	**Influent**	**Post MF**	**Post RO**	**Post UV**	**Sacramento River**	**Colorado River**	**Tucson GW**
**Volume Extracted (ml)**	**250**	**500**	**500**	**500**	**500**	**500**	**250**	**250**	**500**	**500**	**250**	**250**	**250**	**500**	**250**	**500**	**1000**	**1000**	**1000**	**1000**	**1000**
***Pharmaceuticals***																					
Carbamezapine	260	230	230	270	270	260	580	470	460	400	1620	1760	580	590	180	190	<0.5	<0.5	<0.5	1	<0.5
Diclofenac	96	<15	<15	78	34	<15	830	530	420	14	340	370	280	260	120	70	<8	<8	<8	<8	<8
Diltiazem	NA	NA	NA	NA	NA	NA	NA	NA	NA	NA	280	280	130	59	240	140	75	73	<2	<2	<2
Diphenhydramine	1620	1480	1090	280	320	310	25	15	27	<7	<73	<19	<9	<5	470	420	<1	<1	<1	<1	<1
Fluoxetine	160	32	57	41	44	24	79	38	23	34	88	110	89	84	<9	<7	<1	<1	<1	<1	<1
Gemfibrozil	2750	2380	2190	36	38	37	>6000	400	230	190	5550	5300	75	120	680	540	<1	<1	<1	<1	<1
Ibuprofen	>6000	1810	1590	41	41	52	>6000	50	<50	<30	3780	3410	<15	<15	180	120	<15	<15	<15	<15	<15
Meprobamate	1600	650	480	680	670	610	597	421	570	190	690	540	340	280	375	360	<3	<3	<3	3	<3
Naproxen	>6000	550	300	11	10	4	>6000	114	28	6	4740	4170	24	30	970	460	<3	<3	<3	<3	<3
Primidone	200	180	190	170	170	160	1120	620	580	580	370	370	300	300	<20	<8	<4	<4	<4	<4	<3
Sulfamethoxazole	2290	840	1130	1630	1510	990	6080	3910	3010	39	4040	3280	1640	860	590	590	1	1	1	5	<1
Trimethoprim	1110	930	850	130	130	130	1370	30	12	<2	1510	1420	280	200	830	810	<0.5	<0.5	<0.5	1	<0.5
***Personal-care Products***																					
Benzophenone	6300	2320	1710	650	380	300	4540	310	310	220	1670	1640	340	250	880	280	150	130	114	15	21
Bisphenol A	640	140	320	<90	<80	<40	350	20	57	36	240	240	30	<25	69	<20	<20	<20	<20	<20	<20
Caffeine	12000	340	490	6	5	15	6000	22	10	<8	13680	11320	12	12	11	10	<3	<3	4	14	<3
DEET	3570	540	630	46	45	44	2250	190	170	160	700	350	110	130	93	220	<3	<3	5	5	3
Sucralose	32000	23000	15000	15600	14400	13500	9000	8170	7570	7950	23000	21000	19000	19000	25000	23000	38	34	47	620	<31
TCEP	780	690	650	380	350	330	550	330	320	260	630	460	400	370			<3	<3	<3	<3	<3
TCPP	1650	2900	3040	2970	2760	2380	5670	3870	3410	2910	2000	2040	2080	2050	730	1060	<3	<3	11	9	3
Triclocarban	330	31	42	62	96	38	740	200	68	110	580	520	120	9	50	87	<2	<2	<2	<2	<2
Triclosan	2250	440	162	85	71	28	4640	103	69	15	1790	2000	52	29	<39	<13	<6	<6	<6	<6	<6
***Perfluorinated compounds***																					
PFBA	<7	<3	<3	<3	<3	<3	<32	<19	<4	<3	NA	NA	NA	NA	10	9	7	<3	<3	<3	<3
PFBS	17	10	9	13	14	10	24	9	8	5	<5	<4	<3	<3	<3	<2	<1	<1	<1	<1	<1
PFOA	9	7	9	11	45	24	0	46	49	60	<40	<31	<21	<10	<4	<2	<1	<1	<1	<1	<1
PFOS	1080	200	190	5	3	2	460	3	2	4	16	9	10	9	530	290	200	<1	<1	<1	<1
PFDA	<13	<8	<7	<7	<7	<6	<11	<10	<10	<5	<12	<10	<10	<10	<12	<6	<3	<3	<3	<3	<3
PFHxDA	<3	<2	<2	<2	<2	<2	<6	<4	<4	<1	<11	<10	<9	<9	<3	<2	<1	<1	<1	<1	<1
Glucocorticoid																					
Dexamethasone	19	<14	<10	<22	<14	<10	94	<77	<26	<18	<61	<11	<7	<6	<44	<20	<1	<1	<1	<1	<1
Hydrocortisone	<20	<12	<11	<48	<11	<11	NA	<150	<64	<46	<17	<15	<7	<5	<90	<50	<4	<4	<4	7	<5
Prednisone	<165	<61	20	<61	<59	<28	59	NA	NA	NA	NA	<30	<25	<25	<700	<200	<25	<25	<25	<25	<25
Triamcinolone	<55	<23	<21	<27	<21	<21	24	<14	<12	<7	<61	<54	<26	<12	<200	<80	<7	<7	<7	<7	<6
***Pesticides***																					
Atrazine	<10	<9	<2	<2	<2	<2	<20	<3	<2	<2	<3	<2	<2	<1	<19	<6	<1	<1	<1	<1	<1
Simazine	<21	<3	<2	<3	<3	<2	<40	<6	4	3	<6	<4	<2	<2	<38	<12	<2	<2	<2	2	<2
Androgen																					
Testosterone	14	<2	<2	15	14	<2	15	4	3	<2	<11	<8	<5	<2	9	9	<1	<1	<1	<2	<1
***Progestin***																					
Norethistrone	<19	<16	<5	<5	<4	<2	100	6	<5	<5	<30	<7	<3	<2	<18	<7	<3	<3	<3	<3	<3
Norgestrel	93	10	8	18	5	4	19	6	7	3	620	230	<3	<2	110	110	<4	<4	<3	<3	<3

Attached excel file to be placed in main text.

*NA* Not analyzed.

Sucralose (9000–32000 ng/L) and caffeine (6000–13280 ng/L) were present at the highest concentration in the influent of all WWTPs. All pharmaceuticals analyzed in the influent of the three WWTPs were detected with the exception of diphenhydramine in WWTP 3. Concentrations of diabetes and heart-related pharmaceuticals like gemfibrozil, diclofenac, and primidone were significantly higher in the raw sewage of WWTP 2 (the plant serving the dominantly elderly community) compared to the other two WWTPs. Conversely, industrial compounds like benzophenone, PFOS, DEET, and bisphenol A were found at higher concentrations in WWTP 1, potentially confirming the significant industrial input.

The mean effluent concentrations in all WWTPs of artificial sweetener sucralose (13,860) and flame-retardant TCPP (2595 ng/L) were extremely high compared to the other analyzed TOrCs. Their concentrations remained fairly constant throughout the plant indicating that they may be robust and suitable markers for wastewater influence in drinking water sources. Six pharmaceuticals (carbamazepine, gemfibrozil, meprobamate, naproxen, primidone and sulfamethoxazole) were detected in the effluent of all WWTPs with mean concentrations between 85–755 ng/L. Average concentration of sulfamethoxazole (755 ng/L) and gemfibrozil (634 ng/L) were highest in the WWTP effluent for pharmaceuticals. The GR compounds were present at significantly lower concentrations in the influent and not detected in the final effluent in all three WWTPs. However, these compounds still need to be monitored closely as even trace amounts have been shown to have adverse effects to wildlife [8,45]. PFOS was the dominant PFC in terms of detection and concentration while the longer chain PFCs (PFDA and PFHxDA) were not detected at any point in all three WWTPs. PFBS was detected in the effluent of two WWTPs (1 and 2) but at concentration <10 ng/L while PFBA was not detected in any of the effluent samples. Norgestrel was the more frequently detected progestin, present in two effluent WWTP samples (WWTP 1 and 2), while norethisterone was never detected in the effluent. The pesticide atrazine was not detected in any of the samples analyzed throughout the study.

To study the treatment efficacy of the WWTPs, samples were collected at different points in the plant. Further, WWTP 1 had water split into two parallel trains after primary treatment: conventional (activated sludge followed by chlorination) and advanced (advanced air activated sludge, granular media filtration and UV disinfection). The biggest factor in removal of TOrCs between the two treatment trains in WWTP 1 was the type of activated sludge (AS) used. The advanced air activated sludge (AAS) process provided significantly lower concentration of most TOrCs as compared to the AS effluent in the conventional train. The sand filter in WWTP 2 did not have much attenuation of TOrCs, similar to previous literature [46]. Compounds like diclofenac, sulfamethoxazole, naproxen, and triclosan were well removed by the free chlorine disinfection step which is consistent with previously published literature [6]. Conversely, compounds such as DEET, TCPP, TCEP and caffeine are known to be recalcitrant at chlorine doses supplied in conventional treatment plants and hence were not well removed in the chlorination step in both treatment plants. The UV disinfection process (in WWTP 1b) was not very effective in attenuation of TOrCs without the addition of hydrogen peroxide. In DWTP 4, very few TOrCs were attenuated by micro-filtration process, which is consistant with previous literature [47]. However, almost no traces of any TOrCs were detected after the reverse osmosis (RO) process. Only six (benzophenone, diltiazem, PFBA, PFOS, sucralose and sulfamethoxazole) of the 36 measured TOrCs were present after RO treatment in DWTP 4. Of these six, only benzophenone and PFOS were present at concentrations >100 ng/L.

Two surface waters from the Colorado River (sampled at Avra Valley, AZ through the CAP canal) and Sacramento River were analyzed using this method. Eleven target compounds were detected in the Colorado River water while seven were seen in the Sacramento River sample. Six of the target analytes (sucralose, meprobamate, caffeine, DEET, TCPP and benzophenone) were common to both waters. Sucralose was present at the highest concentration in the Colorado River samples at 620 ng/L while in the Sacramento River sample it was measured at 47 ng/L. Commercially used compounds like benzophenone and TCPP were detected at higher concentrations in the Sacramento river while all the other analytes detected were higher in the Colorado River sample. The groundwater sample collected from Tucson had trace amounts of DEET and TCPP (<5 ng/L), and benzophenone at 21 ng/L but all other TOrCs were not detected. Although the sampling events were limited, the data generally correlate with previous studies and hence prove the viability of the analytical method.

## Conclusion

The analytical method presented above allows for rapid, high-throughput detection and quantitation of up to 36 TOrCs including pharmaceuticals, personal care products and steroid hormones using UHPLC-MS/MS. The use of a single all-inclusive SPE method coupled to UHPLC MS/MS provides significant time and labor savings while achieving reporting limits of low ng/L for all analytes. The method has been applied to a wide-range of aqueous matrices. The authors suggest using routine blank analysis, matrix spike recoveries and isotopically-labeled standards for obtaining most accurate results when analyzing different water matrixes.

## Abbreviations

CAV: Cell accelerator voltage; CE: Collision energy; DEET: N,N-Diethyl-meta-toluamide; DWTP: Drinking water treatment plant; ESI: Electrospray ionization; GC: Gas chromatography; GRs: Glucocorticoids; LC: Liquid chromatography; LOD: Limit of detection; LOQ: Limit of quantification; MRL: Method reporting limit; MS: Mass spectrometry; PFBA: Perfluoro butyric acid; PFBS: Perfluoro butane sulfonate; PFC: Polyfluorinated chemical; PFDA: Perfluoro decanoic acid; PFHxDA: Perfluoro hexadecanoic acid; PFOA: Perfluoro octanoic acid; PFOS: Perfluoro octane sulfonate; RO: Reverse osmosis; SPE: Solid-phase extraction; TCEP: Tris (2-chloroethyl) phosphate; TCPP: Tris (2-chloropropyl) phosphate; TOrC: Trace organic contaminant.

## Competing interests

The authors declare that they have no competing interest.

## Authors’ contributions

TA was the primary contributor to this manuscript. TA was responsible for preparing the first draft of the manuscript and performed most of the experimentation and analysis while also being involved heavily in data acquisition and interpretation. SM was involved in design of the experiments and performing some of the data acquisition and analysis. SM also provided critical advice on operation of the analytical equipment due to previous expertise. BOC was involved in conceptualization of the project and did a lot of the initial analytical work. BOC also had a significant role in development of the experiments and interpretation of results. SAS made a considerable intellectual contribution to the development of the study and was chiefly responsible for designing the framework for all the experiments. SAS also had a huge role in producing the finished manuscript and was solely responsible for acquiring funding to purchase all the analytical equipment used in this study. All authors read and approved the final manuscript.

## Supplementary Material

Additional file 1: Table S1 UPLC MS/MS target analytes with mass transitions, compound specific parameters and isotopically labeled surrogate used for quantification. Click here for file

Additional file 2: Table S2 Specifications of UHPLC reverse phase analytical columns tested. Click here for file

Additional file 3: Figure S1 Overlaid EIC traces of the most abundant transition in ESI positive of a methanol blank. The peak at 5.6 min is DEET. Click here for file

Additional file 4: Figure S2 Overlaid EIC traces of the most abundant transition in ESI negative of a methanol blank. Click here for file

Additional file 5: Figure S3 Overlaid EIC of the most abundant transition of DEET in a methanol blank and fortified methanol blank. Click here for file

Additional file 6: Table S3 Summary of treatment processes employed at the three treatment plants included in this study. Click here for file
