# Supplementary material for: Ultra high performance liquid chromatography tandem mass spectrometry for rapid analysis of trace organic contaminants in water
Source: Chem Cent J. 2013 Jun 18;7:104. doi: 10.1186/1752-153X-7-104 (PMC3707776; doi:10.1186/1752-153X-7-104)
Supplement: Additional file 1: Table S1 — UPLC MS/MS target analytes with mass transitions, compound specific parameters and isotopically labeled surrogate used for quantification. [file 1752-153X-7-104-S1.doc]

**Additional file 1 : Table S1.** LC MS/MS target analytes with mass transitions, compound specific parameters and isotopically labeled surrogate used for quantification

| Compound | Precursor Ion (m/z) | Product Ion (m/z) | Fragmentor (V) | Collision Energy (V) | Cell Accelerator Voltage (V) | Retention Time (min) | Isotpoe Used |
| --- | --- | --- | --- | --- | --- | --- | --- |
| **ESI Positive** | | | | | | |  |
| Caffeine | 195.1 | 138 (110.1) | 104 | 16 (24) | 2 | 3.59 | *Caffeine-^13^C_3_* |
| *Caffeine-^13^C_3_* | *198.1* | *140* | *104* | *16* | *2* | *3.59* | *-* |
| Trimethoprim | 291 | 261 (230) | 75 | 25 (25) | 2 | 3.73 | *Trimethoprim-d_3_* |
| *Trimethoprim-d_3_* | *294* | *264* | *75* | *25* | *2* | *3.73* | *-* |
| Sucralose+Na | 419 | 239 (221) | 110 | 15 (15) | 2 | 4.00 | *Sucralose-d_6_* |
| *Sucralose-d_6_* | *425* | *243* | *110* | *15* | *2* | *4.00* | *-* |
| Triamcinolone | 395.2 | 375.1 (225.1) | 95 | 4 (12) | 2 | 4.50 | *Sulfamethoxazole-d_6_* |
| Primidone | 219.3 | 162.1 (91.1) | 70 | 9 (25) | 2 | 4.25 | *Primidone-d_5_* |
| *Primidone-d_5_* | *224* | *167* | *70* | *9* | *2* | *4.25* | *-* |
| Sulfamethoxazole | 254 | 156 (92) | 80 | 10 (30) | 2 | 4.66 | *Sulfamethoxazole-d_6_* |
| *Sulfamethoxazole-d_6_* | *260* | *162* | *80* | *10* | *2* | *4.66* | *-* |
| Meprobamate | 219 | 158 (55) | 70 | 5 (20) | 2 | 4.72 | *Meprobamate-d_7_* |
| *Meprobamate-d_7_* | *226* | *165* | *70* | *5* | *2* | *4.72* | *-* |
| Diphenylhydramine | 256.2 | 167.1 (165.1) | 60 | 4 (44) | 2 | 4.88 | *Sulfamethoxazole-d_6_* |
| Diltiazem | 415.2 | 178 (150) | 130 | 24 (48) | 2 | 4.95 | *Sulfamethoxazole-d_6_* |
| Hydrocortisone | 363.2 | 327 (120.9) | 130 | 13 (24) | 2 | 5.00 | *Sulfamethoxazole-d_6_* |
| Prednisone | 359.2 | 171 (147.1) | 95 | 36 (24) | 2 | 4.98 | *Sulfamethoxazole-d_6_* |
| Simazine | 202.1 | 132 (68.1) | 72 | 16 (36) | 2 | 5.08 | *Atrazine-d_3_* |
| Fluoxetine | 310 | 148 | 90 | 5 | 2 | 5.17 | *Fluoxetine-d_5_* |
| *Fluoxetine-d_5_* | *315* | *153* | *90* | *5* | *2* | *5.17* | *-* |
| Carbamazepine | 237 | 194 (179) | 120 | 15 (35) | 2 | 5.17 | *Carbamazepine-d_10_* |
| *Carbamazepine-d_10_* | *247* | *204* | *120* | *15* | *2* | *5.17* | *-* |
| Dexamethasone | 393.2 | 373.2 (355.2) | 87 | 4 (5) | 2 | 5.22 | *Carbamazepine-d_10_* |
| TCEP | 285 | 222.8 | 95 | 10 | 2 | 5.35 | *Carbamazepine-d_10_* |
| Atrazine | 218 | 176 (174) | 140 | 15 (15) | 2 | 5.49 | *Atrazine-d_3_* |
| *Atrazine-d_3_* | *221* | *179* | *140* | *15* | *2* | *5.49* | *-* |
| DEET | 192 | 119 (91) | 110 | 15 (30) | 2 | 5.53 | *DEET-d_6_* |
| *DEET-d_6_* | *198* | *119* | *110* | *15* | *2* | *5.53* | *-* |
| Testosterone | 289 | 109 (97) | 115 | 25 (25) | 2 | 5.68 | *Carbamazepine-d_10_* |
| Norethistrone | 299.2 | 109.1 (91.1) | 104 | 28 (56) | 2 | 5.75 | *Carbamazepine-d_10_* |
| TCPP | 327 | 99 (81) | 72 | 16 (70) | 2 | 6.20 | *Carbamazepine-d_10_* |
| Norgestrel | 313.2 | 91 (77.1) | 130 | 60 (75) | 2 | 6.18 | *Carbamazepine-d_10_* |
| Benzophenone | 183 | 105.1 | 85 | 10 | 2 | 6.38 | *Carbamazepine-d_10_* |
|  |  |  |  |  |  |  |  |
| **ESI Negative** | | | | | | |  |
| PFOA | 412.9 | 368.9 (169) | 86 | 5 (5) | 7 | 3.03 | *PFOA-^13^C_4_* |
| *PFOA-^13^C_4_* | *416.9* | *371.9* | *86* | *5* | *7* | *3.03* | *-* |
| PFDA | 512.9 | 469 | 102 | 5 | 7 | 3.60 | *PFOA-^13^C_4_* |
| Gemfibrozil | 249.2 | 121 | 75 | 6 | 7 | 3.63 | *Gemfibrozil-d_6_* |
| *Gemfibrozil-d_6_* | *255* | *121* | *75* | *6* | *7* | *3.63* | *-* |
| PFOS | 498.9 | 99 (80) | 210 | 50 (50) | 7 | 3.77 | *PFOS-^13^C_4_* |
| *PFOS-^13^C_4_* | *502.9* | *99* | *210* | *50* | *7* | *3.77* | *-* |
| Triclocarban | 313 | 160 (126) | 110 | 5 (25) | 7 | 4.67 | *Triclocarban-^13^C_6_* |
| *Triclocarban-^13^C_6_* | *318.9* | *159.9* | *110* | *5* | *7* | *4.67* | *-* |
| Triclosan | 289 (287) | 37 (35) | 75 | 5 (5) | 7 | 4.70 | *Triclocarban-^13^C_6_* |
| PFHxDA | 813 | 769 | 100 | 10 | 7 | 5.17 | *PFOA-^13^C_4_* |
| PFBS | 298.8 | 98.9 (80) | 133 | 29 (45) | 7 | 2.42 | *PFOS-^13^C_4_* |
| PFBA | 213 | 169 | 60 | 0 | 7 | 0.97 | *PFBA-^13^C_4_* |
| *PFBA-^13^C_4_* | *217* | *172* | *50* | *5* | *7* | *0.97* | *-* |
| Ibuprofen | 205 | 161 | 50 | 0 | 7 | 2.62 | *Ibuprofen-d_3_* |
| *Ibuprofen-d_3_* | *208* | *164* | *50* | *0* | *7* | *2.62* | *-* |
| Bisphenol A | 227 | 212 (133) | 115 | 11 (19) | 7 | 3.09 | *Bisphenol A-^13^C_12_* |
| *Bisphenol A-^13^C_12_* | *239* | *224* | *115* | *11* | *7* | *3.09* | *-* |
| Naproxen | 229 | 170 (169) | 55 | 4 (24) | 7 | 1.83 | *Naproxen-^13^C_1_d_3_* |
| *Naproxen-^13^C_1_d_3_* | *233* | *169* | *55* | *24* | *7* | *1.83* | *-* |
| Diclofenac | 294 | 250 (214) | 75 | 4 (16) | 7 | 2.52 | *Diclofenac-^13^C_6_* |
| *Diclofenac-^13^C_6_* | *316* | *272.1* | *75* | *5* | *7* | *2.52* | *-* |
|  |  |  |  |  |  |  |  |

()- Qualifier ion.
