# Supplementary material for: Ultra high performance liquid chromatography tandem mass spectrometry for rapid analysis of trace organic contaminants in water
Source: Chem Cent J. 2013 Jun 18;7:104. doi: 10.1186/1752-153X-7-104 (PMC3707776; doi:10.1186/1752-153X-7-104)
Supplement: Additional file 2: Table S2 — Specifications of UHPLC reverse phase analytical columns tested. [file 1752-153X-7-104-S2.doc]

**Additional file 6: Table S2.** Specifications of UHPLC reverse phase analytical columns tested

| **Name** | **Type** | **Dimensions** | **Particle size** | **pH range** |
| --- | --- | --- | --- | --- |
| Zorbax Eclipse Plus | C-18 | 2.1 x 50 mm | 1.8 µm | 2.0-9.0 |
| Zorbax Eclipse Plus | C-8 | 2.1 x 50 mm | 1.8 µm | 2.0-9.0 |
| Zorbax Extend | C-18 | 2.1 x 50 mm | 1.8 µm | 2.0-11.0 |
