# Supplementary figures and images for: Ultra high performance liquid chromatography tandem mass spectrometry for rapid analysis of trace organic contaminants in water
Source: Chem Cent J. 2013 Jun 18;7:104. doi: 10.1186/1752-153X-7-104 (PMC3707776; doi:10.1186/1752-153X-7-104)

## Slide 1
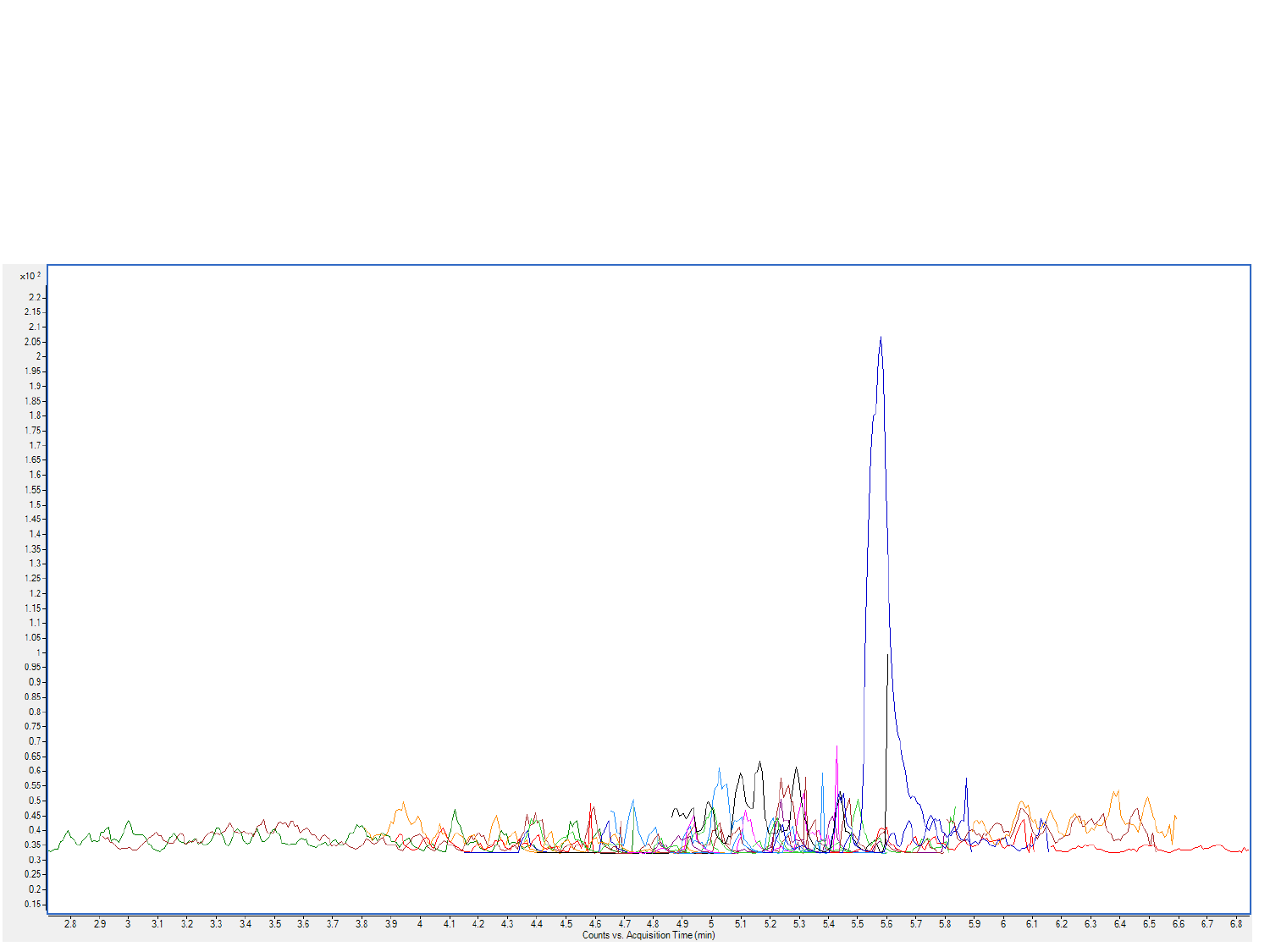

Supplement: Additional file 3: Figure S1 — Overlaid EIC traces of the most abundant transition in ESI positive of a methanol blank. The peak at 5.6 min is DEET. [file 1752-153X-7-104-S3.pptx]

## Slide 1
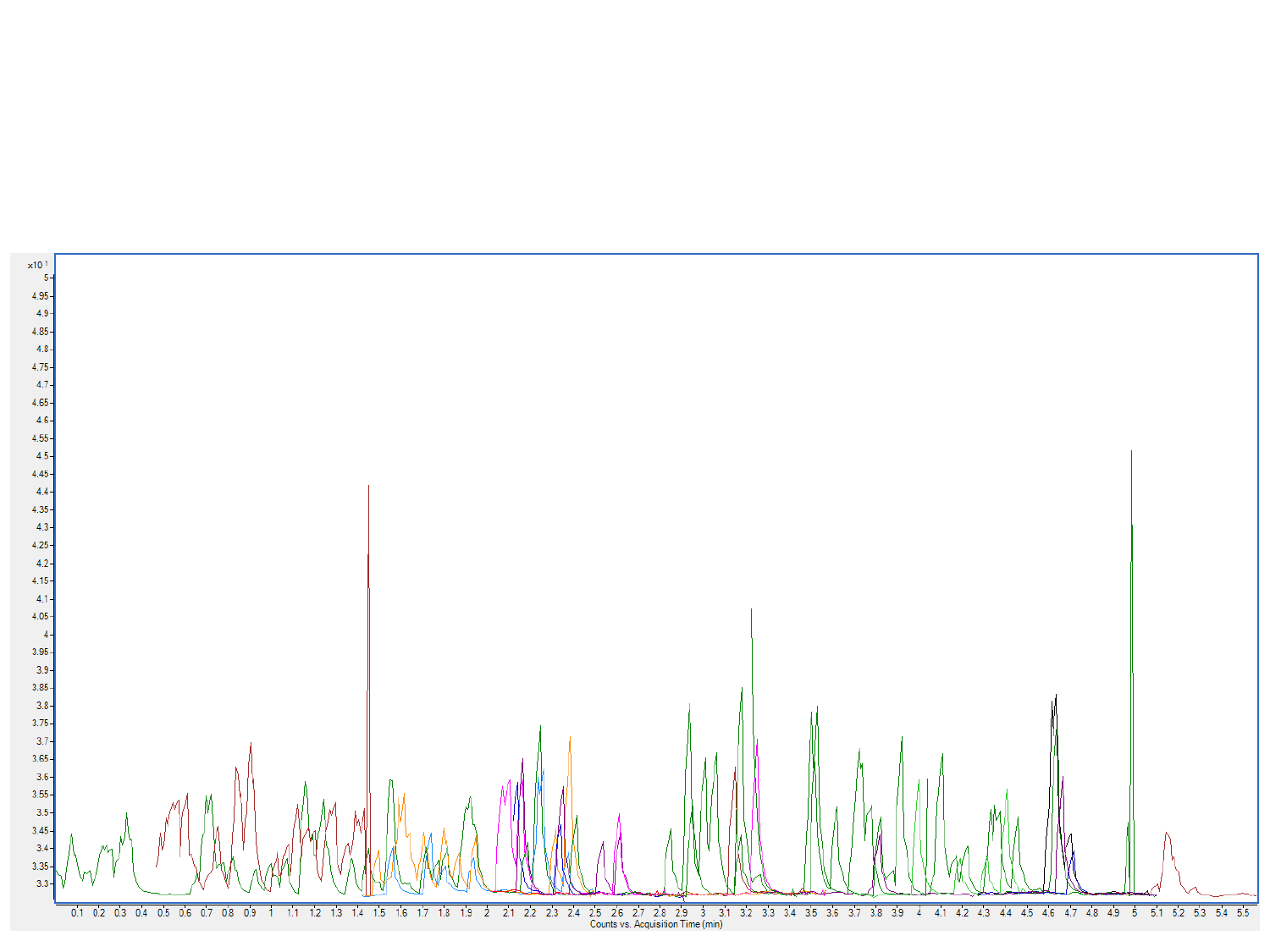

Supplement: Additional file 4: Figure S2 — Overlaid EIC traces of the most abundant transition in ESI negative of a methanol blank. [file 1752-153X-7-104-S4.pptx]

## Slide 1
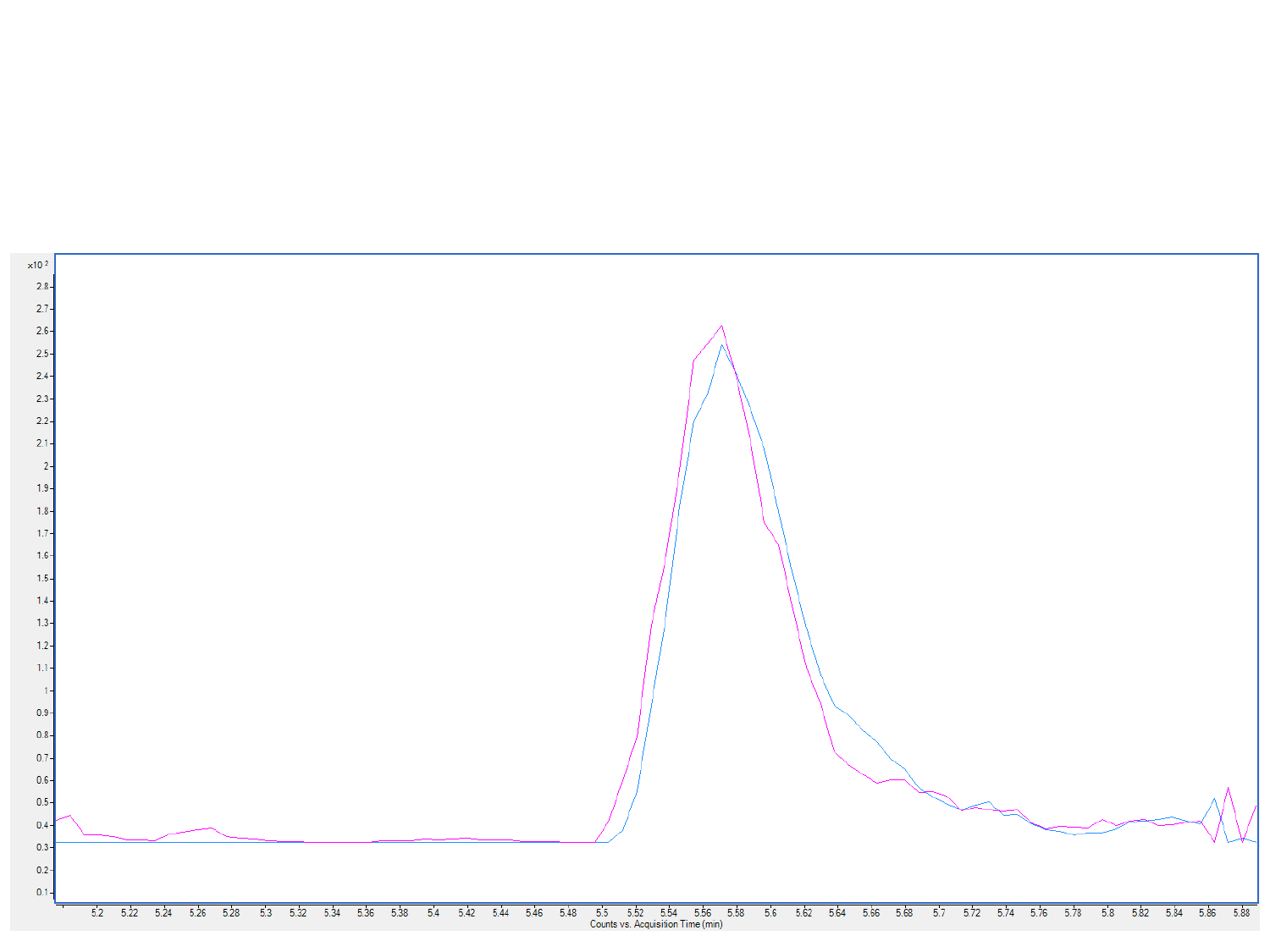

Supplement: Additional file 5: Figure S3 — Overlaid EIC of the most abundant transition of DEET in a methanol blank and fortified methanol blank. [file 1752-153X-7-104-S5.pptx]
