# Supplementary material for: Ultra high performance liquid chromatography tandem mass spectrometry for rapid analysis of trace organic contaminants in water
Source: Chem Cent J. 2013 Jun 18;7:104. doi: 10.1186/1752-153X-7-104 (PMC3707776; doi:10.1186/1752-153X-7-104)
Supplement: Additional file 6: Table S3 — Summary of treatment processes employed at the three treatment plants included in this study. [file 1752-153X-7-104-S6.doc]

**Additional file 5: Table S3.** Summary of treatment processes employed at the three treatment plants included in this study

| WTP | Type | Treatment Scheme |
| --- | --- | --- |
| 1a | Wastewater | Inf→GF→F/S→AS→Cl_2_ |
| 1b | Wastewater | Inf→GF→F/S→AAS→GMF→UV |
| 2 | Wastewater | Inf→BS→BNROD→SF→Cl_2_ |
| 3 | Wastewater | Inf→F/S→AS→Cl_2_ |
| 4 | Drinking Water | Inf→MF→RO→UV |

Inf: Influent; BS: Bar screen; BNROD: Biological nutrient removal with oxidation ditch; SF: sand filter; Cl_2_: Free chlorine disinfection; GF: Grit Filter; F/S: Flocculation/Sedimentation; AS: Activated sludge AAS: Air activated sludge; GMF: Granular media filter; UV: Ultra-Violet light disinfection; MF: Micro-filtration; RO: Reverse Osmosis
